# The organization of the American Sign Language lexicon: Comparing one- and two-parameter ERP phonological priming effects across tasks

**DOI:** 10.1016/j.bandl.2021.104960

**Published:** 2021-04-30

**Authors:** Gabriela Meade, Brittany Lee, Natasja Massa, Phillip J. Holcomb, Katherine J. Midgley, Karen Emmorey

**Affiliations:** a Joint Doctoral Program in Language and Communicative Disorders, San Diego State University, University of California, San Diego, USA; b Department of Psychology, San Diego State University, USA; c School of Speech, Language, and Hearing Sciences, San Diego State University, USA

**Keywords:** Phonological priming, Sign language, ERPs, Task demands

## Abstract

We used phonological priming and ERPs to investigate the organization of the lexicon in American Sign Language. Across go/no-go repetition detection and semantic categorization tasks, targets in related pairs that shared handshape and location elicited smaller N400s than targets in unrelated pairs, indicative of facilitated processing. Handshape-related targets also elicited smaller N400s than unrelated targets, but only in the repetition task. The location priming effect reversed direction across tasks, with slightly *larger* amplitude N400s for targets in related versus unrelated pairs in the semantic task, indicative of interference. These patterns imply that handshape and location play different roles during sign recognition and that there is a hierarchical organization for the sign lexicon. Similar to interactive-activation models of word recognition, we argue for differentiation between sublexical facilitation and lexical competition. Lexical competition is primarily driven by the location parameter and is more engaged when identification of single lexico-semantic entries is required.

## 1. Introduction

Signs, like spoken words, are composed of a discrete set of sublexical phonological units that yield contrastive minimal pairs. The three primary phonological units, or parameters, in sign languages are location, handshape, and movement (for reviews of sign language phonology, see Brentari, 2019; Sandler & Lillo-Martin, 2006). However, surprisingly little is known about how this multi-dimensional phonological structure affects perception and recognition of signs. The investigation of the influence of phonological overlap in priming paradigms has contributed to our understanding of the processes involved in auditory and visual word recognition (for reviews see, e.g., Dufour, 2008; McQueen & Sereno, 2005). Phonological overlap between a prime and a target has also been found to influence sign recognition, and there is some evidence to suggest that the direction of the phonological priming effect may depend on which parameters overlap. However, the literature regarding phonological priming in sign language has not been systematic; drawing conclusions often requires comparisons across studies that differ in terms of stimuli, language, and task, among other important variables. In the present study, we used event-related potentials (ERPs) to clarify the contributions of handshape and location parameters to phonological priming across two tasks that require different levels of lexico-semantic processing. The overall goals of the study were to better understand sign recognition processes and the organization of the sign language lexicon.

An established finding in the auditory priming literature is that different types of phonological overlap tend to yield effects in different directions. Target words preceded by rhyming prime words or pictures (e.g., *cone* – *bone*) are recognized more easily (e.g., have faster lexical decision times) than those preceded by unrelated prime words or pictures (e.g., *lamp* – *bone*; Coch, Grossi, Coffey-Corina, Holcomb, & Neville, 2002; Perre, Midgley, & Ziegler, 2009; Praamstra & Stegeman, 1993). Though less consistent, there is also evidence to suggest that when the prime-target pairs share an onset (e.g., *cone* – *comb*), target words become more difficult to recognize (e.g., Monsell & Hirsh, 1998; Radeau, Morais, & Dewier, 1989; Slowiaczek & Pisoni, 1986). This is especially true as the number of overlapping phonemes increases (e.g., Slowiaczek & Hamburger, 1992). Evidence from ERPs indicates a similar pattern with respect to modulations of the N400 component, a negative peak that occurs about 400 ms after the onset of the target word and is associated with ease of lexico-semantic processing (for a review, see Kutas & Federmeier, 2011). Relative to the unrelated condition, N400 amplitude is smaller for targets in pairs that share a rime, reflecting facilitated processing, and larger for targets in pairs that share an onset, reflecting more effortful processing (Desroches, Newman, & Joanisse, 2009). Just as in the behavioral literature, the electrophysiological signature of onset overlap is comparatively less robust across paradigms and studies (cf. Connolly & Phillips, 1994; Praamstra, Meyer, & Levelt, 1994).

Facilitation from rhyme primes is typically attributed to pre-activation of sublexical phonological representations; the prime word activates phonemes and/or syllables shared with the target before that word is presented, facilitating recognition. In contrast, the inhibitory effect of onset primes can be attributed to lexical competition among co-activated cohort neighbors (i.e., lateral inhibition; Slowiaczek & Hamburger, 1992). Such facilitatory and inhibitory mechanisms can be instantiated within interactive-activation models of word recognition (e.g., McClelland & Elman, 1986; McClelland & Rumelhart, 1981), and we assume that these models can also be applied to sign languages (see also Gutiérrez, Müller, Baus, & Carreiras, 2012).

One of the more important differences between spoken and signed phonological systems is the degree of simultaneity; whereas spoken words consist of sounds presented in sequence, signs consist of a number of visual-manual parameters that are frequently superimposed in time. There is some evidence from the behavioral phonological priming literature to suggest that these various sign phonological parameters also yield different phonological priming effects (e.g., Baus, Gutiérrez, & Carreiras, 2014; Baus, Gutiérrez-Sigut, Quer, & Carreiras, 2008; Carreiras, Gutiérrez-Sigut, Baquero, & Corina, 2008; Corina & Emmorey, 1993; Corina & Knapp, 2006; Dye & Shih, 2006; Mayberry & Witcher, 2005). For example, location overlap between pairs of signs typically slows lexical decision reaction times relative to a phonologically unrelated control condition (e.g., Carreiras et al., 2008; Corina & Emmorey, 1993), but neither handshape nor movement overlap affect reaction times in a reliable way across studies (e.g., Carreiras et al., 2008; Corina & Emmorey, 1993; Dye & Shih, 2006). Moreover, when both location *and* another parameter overlap, the interference often disappears (Baus et al., 2014; Corina & Knapp, 2006; Dye & Shih, 2006). Recent ERP investigations of phonological priming in sign language provide preliminary support for this dissociation between two-parameter overlap resulting in facilitation and location-only overlap resulting in interference. In a semantic relatedness judgment task, target signs elicited smaller amplitude N400s when preceded by primes that shared any two parameters relative to unrelated primes (e.g., Meade, Lee, Midgley, Holcomb, & Emmorey, 2018; see also Meade, Midgley, Sevcikova Sehyr, Holcomb, & Emmorey, 2017). In a lexical decision task, target signs elicited *larger* amplitude N400s when preceded by primes that shared only location relative to unrelated primes, and there was no significant effect of handshape overlap (Gutiérrez et al., 2012). These varied results indicate that a comprehensive account of sign processing involves special consideration of the role that each parameter might play in sign recognition.

More specifically, identifying what sets location apart from the other parameters is paramount to understanding the organization of the sign lexicon and the processes that underlie sign recognition. Some authors have argued that location holds a special status at the lexical level. The interference caused by location overlap has been suggested to reflect an increase in lexical competition when the prime and target are location neighbors and inhibit one another (e.g., Carreiras et al., 2008). Gutiérrez et al. (2012) went further to draw a parallel between location overlap in sign language and onset overlap in studies of spoken word recognition. Others have argued that location holds a special status at the level of sublexical representations. Based on simulations using a computational model, Caselli and Cohen-Goldberg (2014) argued that the difference between location and handshape occurs at the sublexical level rather than at the lexical level. They failed to simulate opposing priming effects for location versus handshape by manipulating the number of lexical neighbors. Low amounts of activation generated facilitation and high amounts of activation generated inhibition, with the respective effects increased for signs with more neighbors. However, they were able to model the empirical pattern of results by modulating two other parameters. When location information was made available to the model before handshape, the inhibitory effects for location neighbors emerged. This finding mirrors empirical evidence from a gating paradigm suggesting that the location parameter might be perceived earlier than other parameters (see Emmorey & Corina, 1990). Inhibitory effects for location neighbors also emerged when the resting level of activation differed for the two parameters (i.e., higher for location than handshape), which Caselli and Goldberg-Cohen associated with sublexical frequency. On the basis of these simulations, the authors concluded that processing of the two parameters diverges because of “variation in the activation of sub-lexical units rather than lexical units” (p. 9), but acknowledged that more empirical research is needed.

By comparing the ERP priming effects elicited by various types of phonological overlap within the same group of deaf signers across two different tasks, we lend insight into sign processing at the sublexical and lexical levels. Prime and target signs overlapped in location only, handshape only, or both handshape and location. We chose to focus on these two parameters since they were included in the model by Caselli and Goldberg (2014) and seem to yield priming effects in opposite directions. We predicted that target signs preceded by primes that overlapped in two phonological parameters would be easier to process and recognize than those preceded by unrelated primes, as reflected in smaller amplitude N400s for targets in the related prime condition (e.g., Meade, Lee, et al., 2018; Meade et al., 2017). We also expected either an effect of priming in the same direction or no effect at all for target signs preceded by primes that overlapped in handshape only (cf. Gutiérrez et al., 2012). However, the effect of location-only priming was expected to go in the opposite direction (i.e., larger amplitude N400s for targets preceded by related primes) based on previous ERP data (Gutiérrez et al., 2012) and relatively consistent findings of interference in the behavioral literature (Carreiras et al., 2008; Corina & Emmorey, 1993; but see Dye & Shih, 2006). Finding that targets preceded by location-only related primes elicit *larger* amplitude N400s than those preceded by unrelated primes with all else held equal would establish the special status of that parameter.

In order to better understand the level at which location might interfere with processing, we compared the size and direction of these various phonological priming effects presenting the same stimuli across tasks that require a different “depth” of processing. Following a rich tradition in the ERP literature, adjusting the task demands was assumed to modulate the emphasis on different levels of processing (e.g., Chwilla, Brown, & Hagoort, 1995; Meade, Grainger, & Holcomb, 2019; Ziegler, Besson, Jacobs, Nazir, & Carr, 1997). To orient participants toward deeper lexico-semantic processing, we chose a go/no-go semantic categorization task in which participants identified occasional target signs that were names of countries (e.g., FLOWER-AUSTRALIA). To orient participants toward shallower form processing, we chose a go/no-go repetition detection task in which participants pressed a button when the target sign was the same as the prime (e.g., FLOWER-FLOWER). If the “reversed” location priming effect described above is in fact due to lexical competition, then it should be more prominent in the semantic task that requires identification of specific signs. The repetition detection task places less emphasis on identification of the meaning of specific signs and, as a result, does not rely on competition among lexical neighbors to the same extent. Thus, the reversed N400 location priming effect would not be expected in the repetition task. In contrast, if the location interference is due to sublexical characteristics (e.g., timing, frequency) then the priming effect should go in the same inhibitory direction across both tasks. Even though we predicted the direction of the priming effect to go in the same direction across tasks for the handshape-only and two-parameter overlap conditions, we expected the magnitude of the effects to differ across the repetition detection and semantic categorization tasks. Following the word rhyme literature (e.g., Yoncheva, Maurer, Zevin, & McCandliss, 2013, 2014), we predicted that the priming effects in ASL would be larger in the repetition detection task in which participants’ attention was explicitly drawn toward the surface characteristics of the signs, including phonology. Taken together, these various comparisons will refine our understanding of the organization of the lexicon, and the mechanisms underlying phonological priming in sign languages.

## 2. Methods

### 2.1. Participants

Data from 20 severely-to-profoundly deaf individuals (10 female; mean age = 32.8 years; *SD* 7.1 years) are presented. Four of these participants reported being left-handed and the remaining 16 reported being right-handed. All participants began learning ASL before the age of seven (four from birth) and had normal or corrected-to-normal vision. All participants provided consent in accordance with the Institutional Review Board at San Diego State University. Two additional participants who met the same criteria and participated in both tasks were excluded due to high artifact rejection rates (>25%) or experimenter error.

### 2.2. Stimuli

**Fig. 1.**
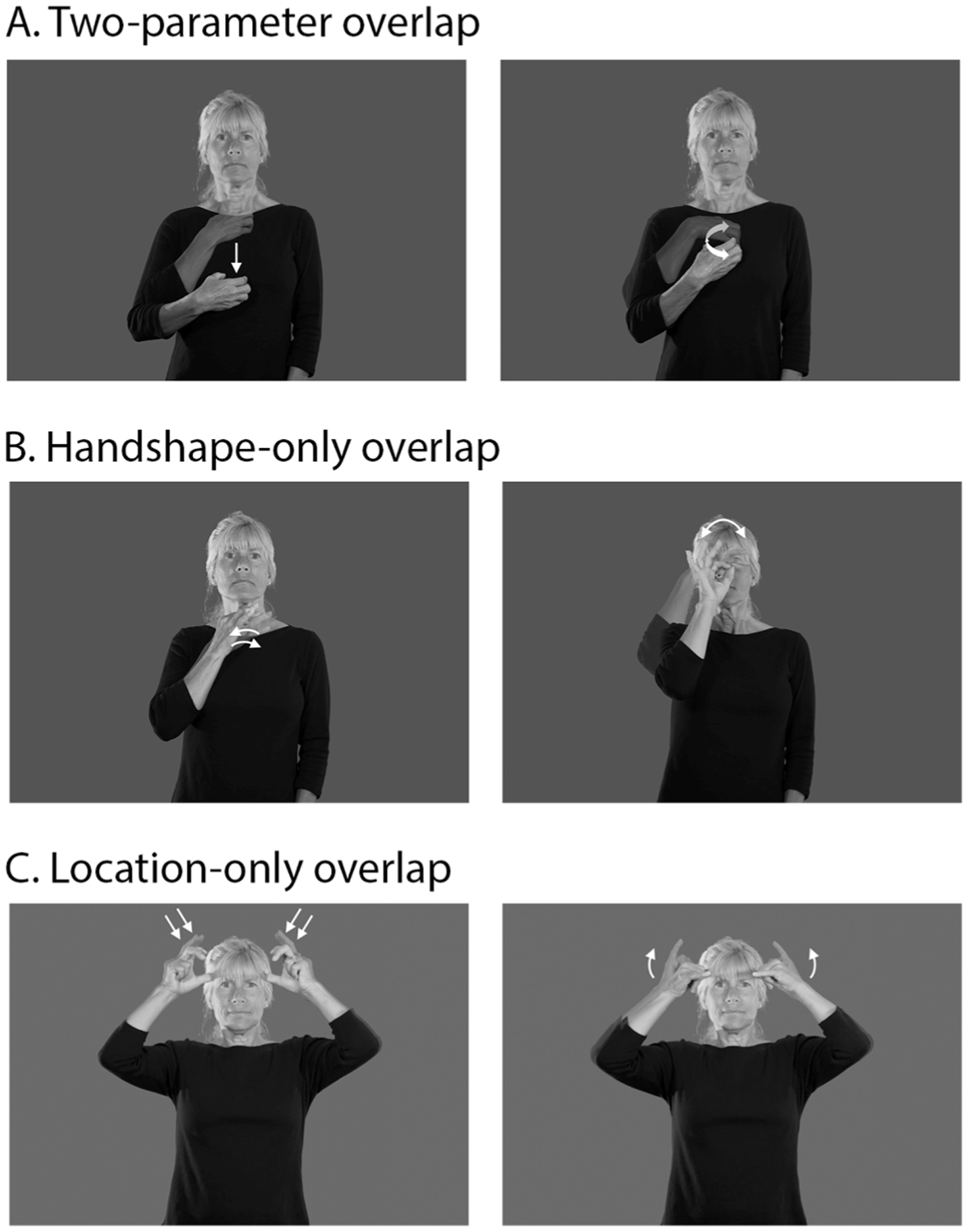
Example stimuli. The ASL signs HUNGRY and COUGH share both handshape and location, but differ in movement (A), whereas the ASL signs CURIOUS and FOX share only handshape (B) and the ASL signs DEVIL and COW share only location (C).

The full list of stimuli is available at https://osf.io/w7pj4/; using the Entry IDs (glosses) listed, videos of all but nine critical signs can be found in the ASL-LEX database (asl-lex.org) or in Signbank (aslsignbank.haskins.yale.edu). Critical stimuli consisted of 138 ASL sign triplets (e.g., HUNGRY, SEE, COUGH), which were each used to form two pairs with the same target (e.g., HUNGRY-COUGH, SEE-COUGH). One of the pairs in each triplet was phonologically related (e.g., HUNGRY and COUGH have the same “C” handshape and location on the chest; see Fig. 1) and the other was phonologically unrelated (e.g., SEE and COUGH do not overlap in any phonological parameters). Phonologically related sign pairs fell into one of three conditions with 46 targets per condition: handshape-only overlap, location-only overlap, and two-parameter (i.e., handshape and location) overlap. None of the related prime-target pairs overlapped in movement and none of the unrelated pairs overlapped in any of the three phonological parameters. In general, variants of a given handshape were considered to be phonologically distinct forms (e.g., O and flat O), but every effort was made to ensure that unrelated primes had completely distinct handshapes rather than variants of the same handshape. Signs were classified as having a location in neutral space or at one of the ‘minor’ body locations specified in Sehyr, Caselli, Cohen-Goldberg, and Emmorey (2021) based on Brentari (1998): top of the head, forehead, side of head (head away), eye, cheek/nose, mouth, chin, under the chin, neck, shoulder, torso-top, torso-bottom, upper arm, forearm front. Signs produced on the nondominant hand in neutral space (e.g., HELP, COOK) were excluded to avoid ambiguity about their location.

An additional 46 pairs were included as probe targets for the go/no-go tasks but were not analyzed. These pairs consisted of two different exemplars of the same sign (e.g., FLOWER-FLOWER) in the repetition detection task and the same primes followed by country signs (e.g., FLOWER-AUSTRALIA) in the semantic categorization task. We were only able to identify 31 commonly used country signs in ASL so a subset of 15 were randomly selected to be repeated once to obtain a total of 46 probe target items.

A native ASL signer was filmed producing each of the signs at a natural rate without mouthing. Each video was clipped to begin two frames before sign onset and end at sign offset. Sign onsets and offsets were determined as in previous studies (see, e.g., Caselli, Sevcikova Sehyr, Cohen-Goldberg, & Emmorey, 2017; Meade, Lee, et al., 2018). Two fluent L2 hearing signers coded onsets and offsets for a randomly selected 20% of the stimuli with high interrater reliability (96% agreement for onsets and 83% agreement for offsets within a three-frame margin) before coding the remaining 80%.

### 2.3. Procedure

Each trial consisted of a prime sign followed by a target sign presented in ASL. As in our previous ASL phonological priming study (Meade, Lee, et al., 2018), the sign pairs were separated by a 1300 ms stimulus onset asynchrony that consisted of the prime video and an interstimulus interval (ISI) of variable duration. A grey rectangle that subtended a visual angle of 10.8° in the vertical direction and 14.0° in the horizontal direction remained at the center of the black screen for the duration of each trial. The model appeared within the grey rectangle, subtending a visual angle of 9.7° in the vertical direction and 4.9° in the horizontal direction. She signed the prime, disappeared during the ISI, and reappeared to sign the target. A black screen appeared immediately after the target video and remained on the screen for 800 ms to minimize artifacts during the epoch of interest. A purple fixation cross then appeared at the center of the black screen for 1500 ms, during which participants were instructed to blink. It was replaced by a white fixation cross for 500 ms and a blank screen for 500 ms before the beginning of the next trial. Trials were arranged in a pseudorandomized order with longer blink breaks approximately every 20 trials. Two lists were formed such that each target occurred twice in each list, preceded by a phonologically related prime in one half of the list and by a phonologically unrelated prime in the other half. One list was the reverse order of the other, and list order was counterbalanced across participants within each task.

Participants completed the two tasks at least one month apart. The instructions for the two tasks were as similar as possible and were provided in both written English and ASL. In the repetition task, participants were instructed to press a button when the first and second signs in a pair were the exact same sign. In the semantic categorization task, they were instructed to press when they saw a country target sign. Before both tasks, participants were explicitly told that they would be seeing signs that overlap in location, movement, and/or handshape. Each task began with a practice list that contained eight sign pairs, two of which were probes and none of which occurred during the main experiment.

### 2.4. EEG recording and ERP analysis

EEG was recorded from 29 active electrodes in an Electro-Cap. Additional electrodes were placed beside the outer canthus of the right eye to monitor for horizontal eye movement, below the left eye to monitor for blinks in conjunction with electrodes on the forehead, and on both mastoids. Scalp and mastoid electrode impedances were maintained below 2.5 kΩ and eye electrodes below 5 kΩ. EEG was amplified by a SynAmpsRT amplifier (Neuroscan-Compumedics) with a bandpass of DC to 100 Hz and was continuously sampled at 500 Hz.

ERPs time-locked to target video onset and referenced to the left mastoid were averaged separately for each condition and processed with a 15 Hz low-pass filter. Trials contaminated by eye movement or drift artifact during the 100 ms pre-target-onset baseline or within 900 ms of target video onset were rejected prior to averaging, as were critical trials with button presses (i.e., false alarms). An average of 20 critical trials (7%) contained artifacts in the semantic categorization task and 26 critical trials (10%) in the repetition detection task. A repeated-measures ANOVA including factors Task (semantic categorization, repetition detection), Parameter (HS, LOC, HS + LOC), and Prime (related, unrelated) revealed no significant differences in the rejection rates across conditions, all *p*s > 0.10 (see Table 1).

**Fig. 2.**
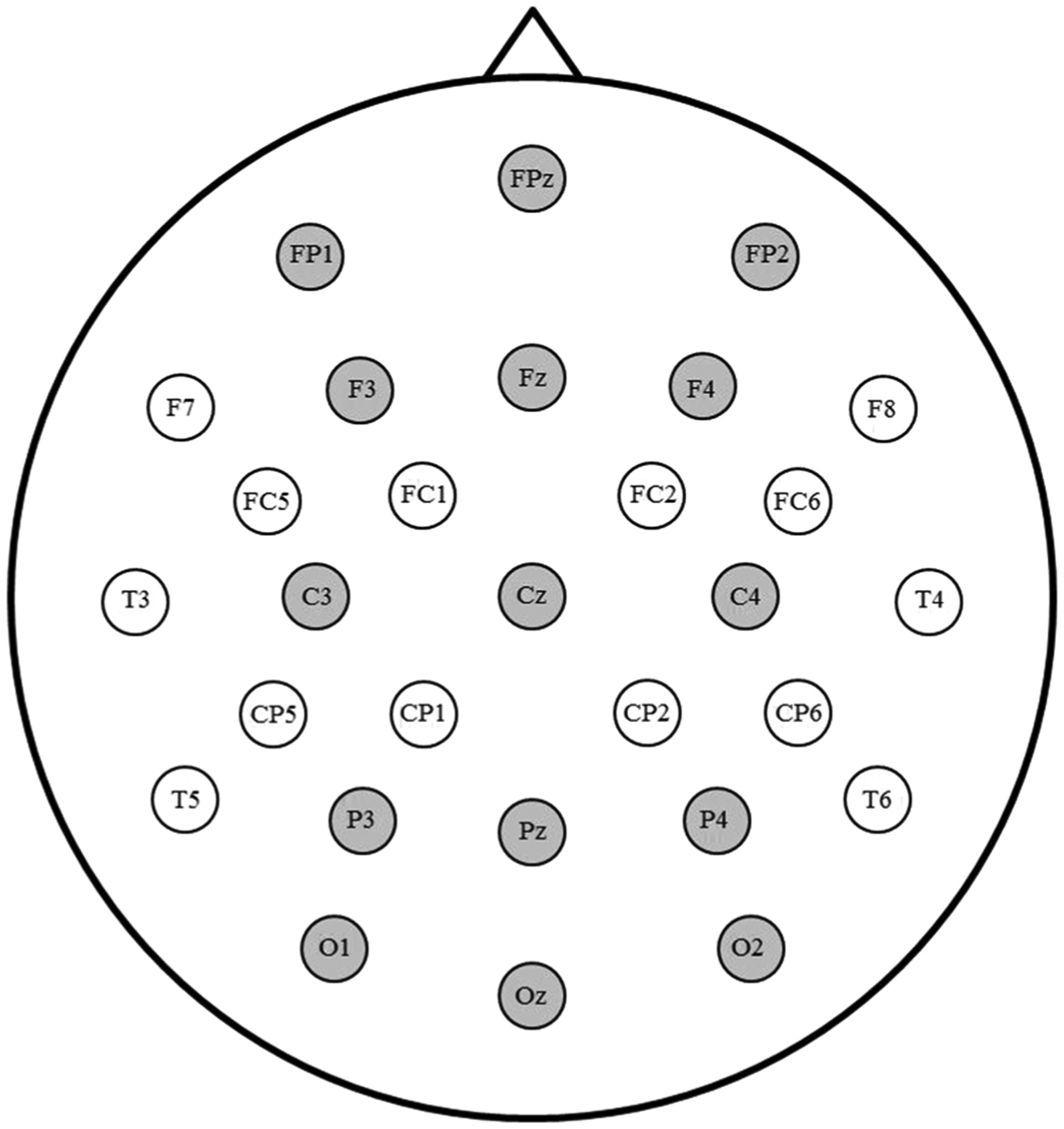
Electrode montage. The 15 sites included in analyses are highlighted in grey.

Based on visual inspection of the grand average waveforms, mean N400 amplitude was calculated between 400 and 600 ms. Repeated measures analyses of variance (ANOVAs) were used to analyze mean N400 amplitude at the 15 electrode sites illustrated in Fig. 2. Given evidence that phonological priming effects in ASL do not have the classic centro-parietal distribution observed for semantic priming N400 effects (e.g., Meade, Lee, et al., 2018), we opted for an array that we have found to provide good coverage of the scalp in previous studies (e.g., Lee, Meade, Midgley, Holcomb, & Emmorey, 2019). Separate ANOVAs were conducted for the three types of phonological relatedness. Each included within-participant factors: Task (Semantic, Repetition), Prime (Related, Unrelated), Laterality (Left, Midline, Right) and Anterior/Posterior (Prefrontal, Frontal, Central, Parietal, Occipital). To understand significant interactions that included both Task and Prime, we analyzed the priming effect separately in each task. All significant results (i.e., *p* < .05) from these analyses are reported below.^1^ Partial eta squared $\left(\eta_{\text{p}}^{2}\right)$ is reported as a measure of effect size. Greenhouse-Geisser correction was applied to all effects with more than one degree of freedom in the numerator.

## 3. Results

### 3.1. Behavioral performance

Participants identified an average of 43.2 (*SD* 3.4) of the 46 probes in the repetition detection task and 41.2 (*SD* 3.9) of the 46 probes in the semantic categorization task, a difference that just failed to reach significance, *F*(1,19) = 4.19, *p* = .055, $\eta_{\text{p}}^{2}=0.18$. Excluding trials with reaction times more than two standard deviations away from the mean for each individual participant in each task, reaction times were significantly faster in the repetition detection task (mean 853 ms, *SD* 146 ms) than in the semantic categorization task (mean 972 ms, *SD* 140 ms), *F*(1,19) = 14.14, *p* = .001, $\eta_{\text{p}}^{2}=0.43$. Together, these patterns confirm that participants were attentive to the signs during both tasks and suggest that the semantic categorization task may have been more challenging or required additional time to allow for processing of the entire sign.

We also analyzed the distribution of false alarms across conditions in each task; that is, the number of critical trials that elicited erroneous button presses (see Table 2). In the repetition detection task, there was a significant main effect of Prime, such that related trials elicited more false alarms (i.e., repetition responses) than unrelated trials, *F*(1,19) = 7.98, *p* = .011, $\eta_{\text{p}}^{2}=0.29$. A significant main effect of Parameter further indicated that the false alarm rate differed for targets across the various conditions, *F*(2,38) = 7.43, *p* = .007, $\eta_{\text{p}}^{2}=0.28$. Finally, a significant Prime × Parameter interaction indicated that the effect of prime relatedness on the false alarm rate differed for the various parameters, *F* (2,38) = 6.54, *p* = .014, $\eta_{\text{p}}^{2}=0.26$. In follow-up analyses by parameter, the effect of prime relatedness was only significant for HS + LOC targets, such that HS + LOC targets preceded by related primes were more likely to elicit false alarm responses than those preceded by unrelated primes, *F*(1,19) = 7.73, *p* = .012, $\eta_{\text{p}}^{2}=0.29$. In the semantic categorization task, the phonological relatedness between the prime and target did not influence false alarm rates overall, Prime, *F*(1,19) = 1.95, *p* = .179, $\eta_{\text{p}}^{2}=0.09$. However, the main effect of Parameter was significant, *F*(2, 38) = 8.95, *p* = .001, $\eta_{\text{p}}^{2}=0.32$, as was the Prime × Parameter interaction, *F*(2, 38) = 4.74, *p* = .015, $\eta_{\text{p}}^{2}=0.20$. Follow-up analyses indicated that targets in the HS related condition elicited more false alarms than those in the unrelated condition, *F*(1,19) = 5.38, *p* = .032, $\eta_{\text{p}}^{2}=0.22$, whereas the effect went in the opposite direction in the LOC condition, *F*(1,19) = 5.90, *p* = .025, $\eta_{\text{p}}^{2}=0.24$. It is unclear why phonological similarity would have any effect on false alarm rates in the semantic categorization task, much less why these effects would go in opposite directions for different parameters, so we do not consider these results further.

### 3.2. N400

**Fig. 3.**
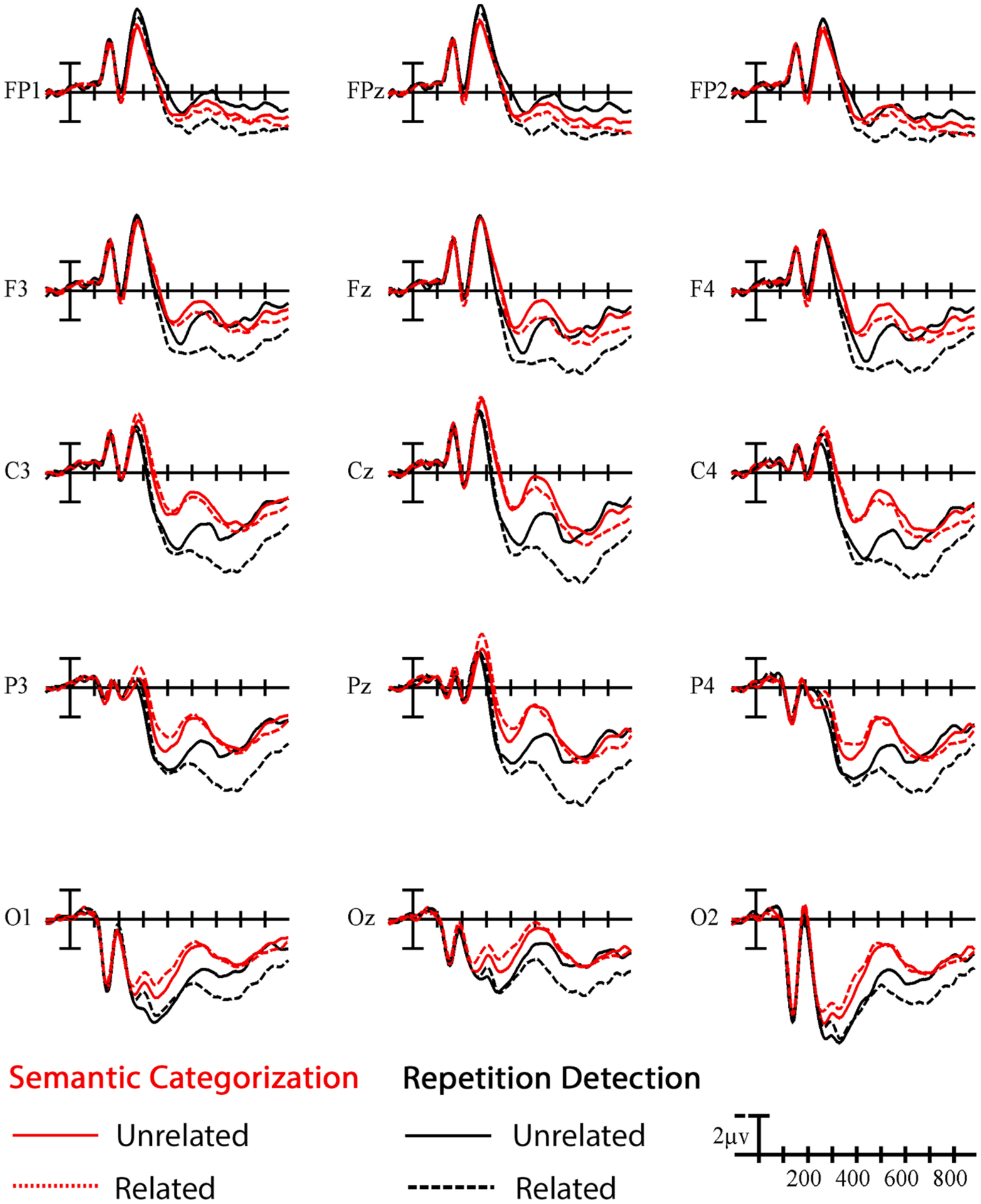
Task and priming effects for all critical trials. Grand average ERP waveforms elicited by all target signs in the related (dotted) and unrelated (solid) conditions in both the repetition detection (black) and semantic categorization (red) tasks. Each vertical tick marks 100 ms and negative is plotted up. The calibration bar marks 2 μV.

To provide a general overview of the results, ERPs illustrating the main effect of priming (i.e., collapsed across the three overlap conditions) for each task are presented in Fig. 3. Given that different target lexical items occurred across the three parameter conditions, we analyzed the effect of phonological priming separately for each parameter condition.

#### Two-parameter overlap.

**Fig. 4.**
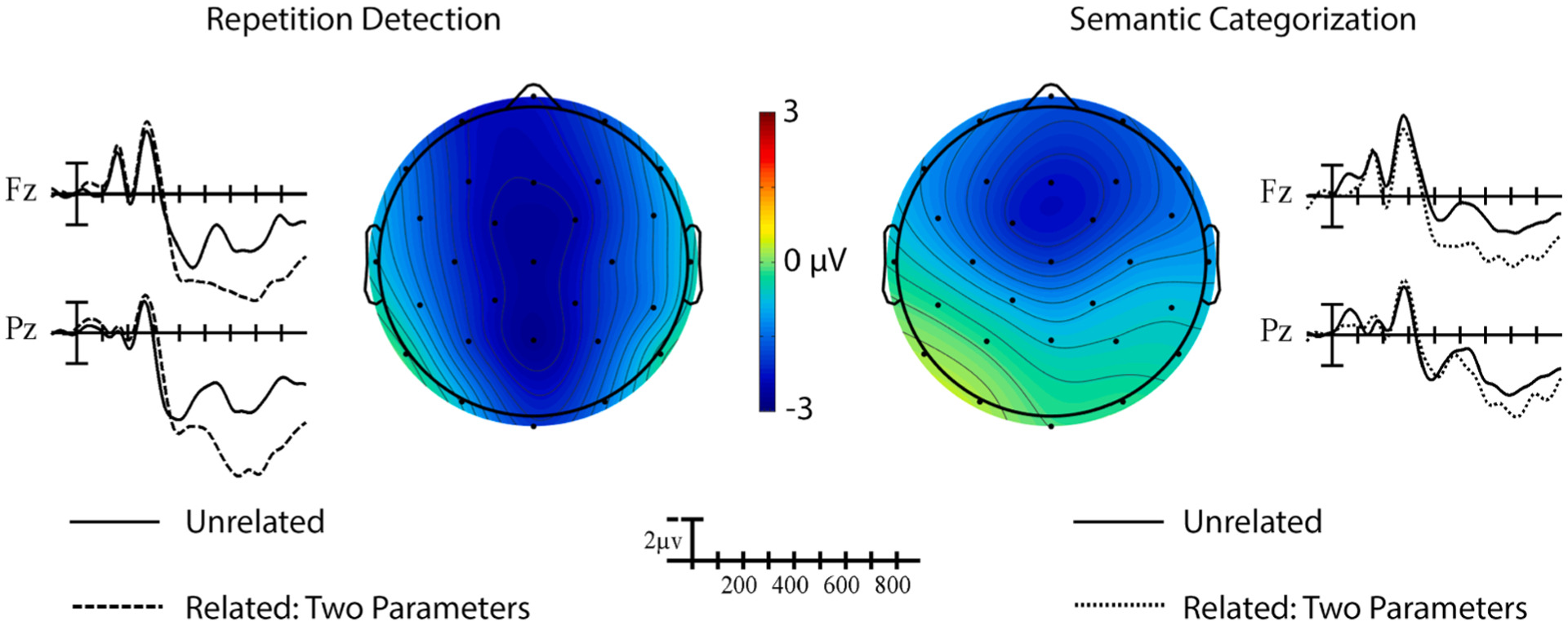
Two-parameter priming effects. Grand average ERP waveforms elicited by target signs in the two-parameter condition preceded by related (dotted) and unrelated (solid) prime signs across the two tasks at representative sites Fz and Pz. Each vertical tick marks 100 ms and negative is plotted up. The calibration bar marks 2 μV. The scalp voltage maps show the distribution of the priming effects in the respective tasks in the measured N400 window (unrelated-related).

A significant main effect of Prime indicated that targets preceded by phonologically related primes that shared two parameters elicited smaller amplitude N400s than those preceded by unrelated primes (see Fig. 4), Prime, *F*(1,19) = 19.17, *p* < .001, $\eta_{\text{p}}^{2}=0.50$. This difference was strongest at midline and anterior sites, Prime × Laterality, *F*(2,38) = 8.67, *p* = .003, $\eta_{\text{p}}^{2}=0.31$, Prime × Anterior/Posterior, *F*(4,76) = 6.14, *p* = .009, $\eta_{\text{p}}^{2}=0.24$. A significant main effect of Task indicated that targets in the semantic task elicited larger amplitude N400s than targets in the repetition detection task (cf. Fig. 3), Task, *F*(1,19) = 28.26, *p* < .001, $\eta_{\text{p}}^{2}=0.60$. The effect of task was strongest around the apex of the head, Task × Anterior/Posterior, *F* (4,76) = 8.45, *p* = .001, $\eta_{\text{p}}^{2}=0.31$, Task × Laterality × Anterior/Posterior, *F*(8,152) = 2.86, *p* = .045, $\eta_{\text{p}}^{2}=0.12$. Visually, the phonological priming effect appeared to be bigger and more widespread in the repetition task compared to the semantic task, but the interactions between Prime and Task did not reach significance, all *p*s > 0.08.

#### Handshape-only overlap.

**Fig. 5.**
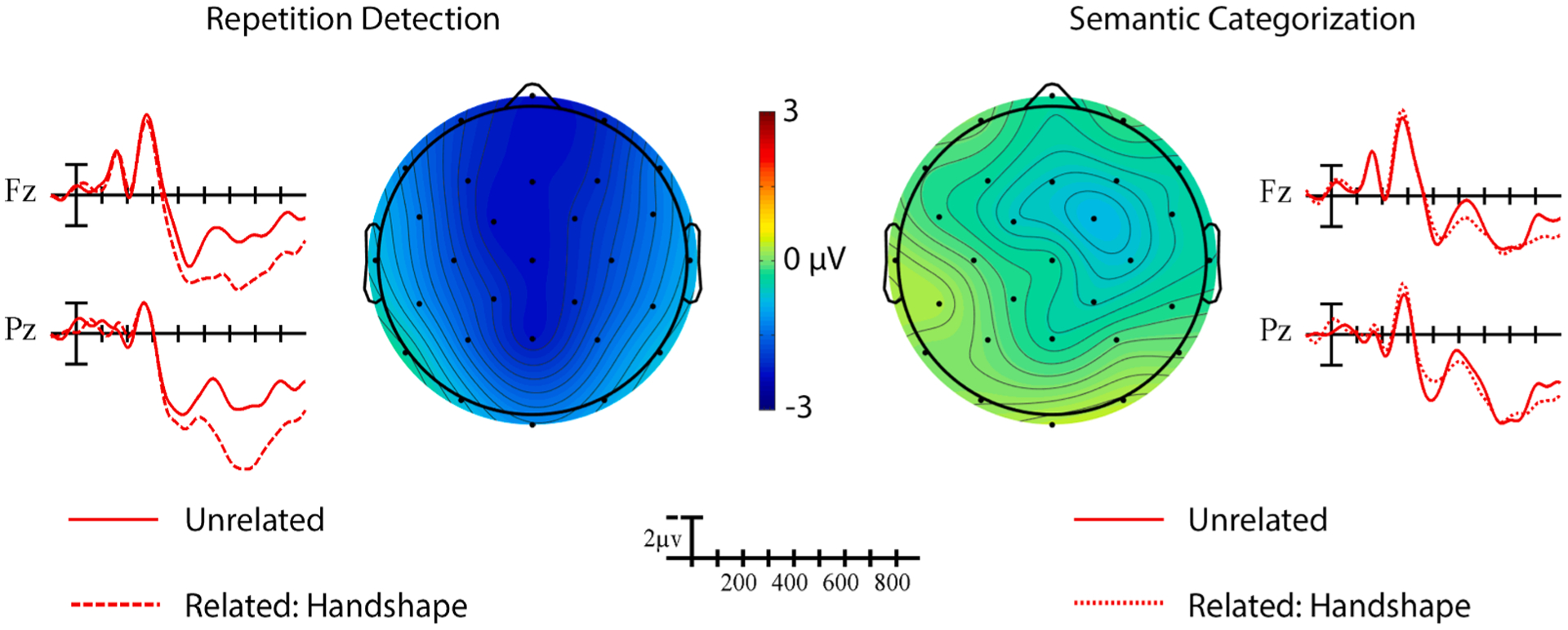
Handshape priming effects. Grand average ERP waveforms elicited by target signs in the handshape-only condition preceded by related (dotted) and unrelated (solid) prime signs across the two tasks at representative sites Fz and Pz. Each vertical tick marks 100 ms and negative is plotted up. The calibration bar marks 2 μV. The scalp voltage maps show the distribution of the priming effects in the respective tasks in the measured N400 window (unrelated-related).

A significant main effect of Prime indicated that targets preceded by phonologically related primes that only shared handshape also elicited smaller amplitude N400s than those preceded by unrelated primes (see Fig. 5), Prime, *F*(1,19) = 12.39, *p* = .002, $\eta_{\text{p}}^{2}=0.39$. The overall handshape priming effect was strongest at midline sites, Prime × Laterality, *F*(2,38) = 6.08, *p* = .011, $\eta_{\text{p}}^{2}=0.24$. A significant main effect of Task further indicated that targets in the semantic task elicited larger amplitude N400s than targets in the repetition task (cf. Fig. 3), Task, *F*(1,19) = 31.94, *p* < .001, $\eta_{\text{p}}^{2}=0.63$. The effect of task was strongest at central right hemisphere sites, Task × Laterality, *F*(2,38) = 3.73, *p* = .035, $\eta_{\text{p}}^{2}=0.16$, Task × Anterior/Posterior, *F*(4,76) = 6.12, *p* = .008, $\eta_{\text{p}}^{2}=0.24$, Task × Laterality × Anterior/Posterior, *F*(8,152) = 3.21, *p* = .033, $\eta_{\text{p}}^{2}=0.14$. However, contrary to the two-parameter priming effect, the size of the handshape priming effect was significantly modulated by task, Prime × Task, *F*(1,19) = 8.73, *p* = .008, $\eta_{\text{p}}^{2}=0.31$. In the repetition task, there was a widespread effect of handshape priming such that targets in phonologically related pairs elicited smaller amplitude N400s than targets in unrelated pairs, *F* (1,19) = 25.96, *p* < .001, $\eta_{\text{p}}^{2}=0.58$. In contrast, there were no significant effects of handshape priming in the semantic task, all *p*s > 0.29.

#### Location-only overlap.

**Fig. 6.**
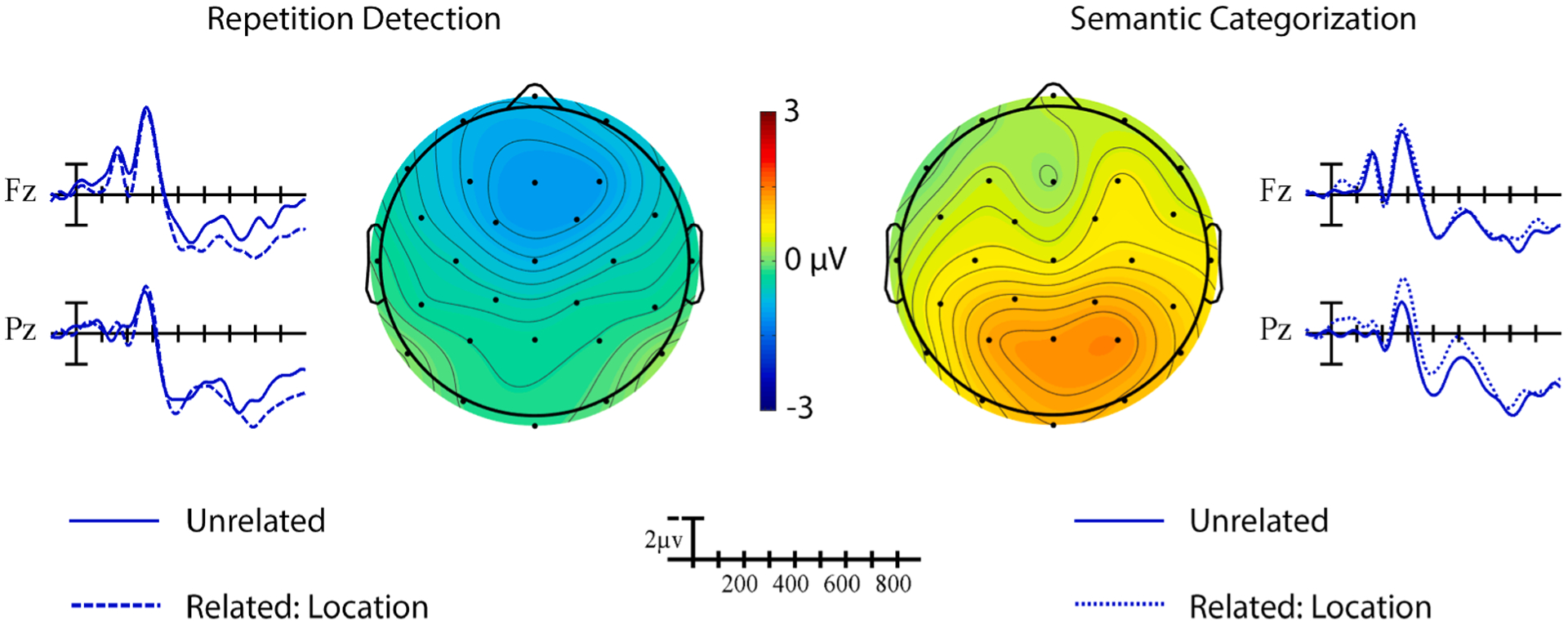
Location priming effects. Grand average ERP waveforms elicited by target signs in the location-only condition preceded by related (dotted) and unrelated (solid) prime signs across the two tasks at representative sites Fz and Pz. Each vertical tick marks 100 ms and negative is plotted up. The calibration bar marks 2 μV. The scalp voltage maps show the distribution of the priming effects in the respective tasks in the measured N400 window (unrelated-related).

In contrast to the previous analyses, there were no significant effects of location priming overall, all *p*s > 0.05. This result may have occurred because the direction of the ERP priming effect reversed across tasks (see Fig. 6), Prime × Task, *F*(1,19) = 5.55, *p* = .029, $\eta_{\text{p}}^{2}=0.23$. In the repetition task, targets in location related pairs elicited *smaller* amplitude N400s than those in unrelated pairs, whereas the opposite pattern was observed in the semantic task. However, neither of these priming effects reached significance in follow-up analyses by task, all *p*s > 0.15. A significant effect of Task in the omnibus analysis indicated that targets in the semantic task elicited larger amplitude N400s than those in the repetition task (cf. Fig. 3), Task, *F* (1,19) = 12.77, *p* = .002, $\eta_{\text{p}}^{2}=0.40$. The effect of task was larger across central sites, Task × Anterior/Posterior, *F*(4,76) = 10.13, *p* = .001, $\eta_{\text{p}}^{2}=0.35$.

## 4. Discussion

The goal of the present study was to improve our understanding of how the sign language lexicon is organized and to delineate the layered processes that unfold during sign recognition. To achieve this, we investigated how the type and degree of phonological overlap influences sign recognition in the context of two tasks that require different levels of lexico-semantic processing. Specifically, we presented the same pairs of ASL signs to the same participants in go/no-go repetition detection and semantic categorization tasks. Critical sign pairs were either unrelated or fell into one of three phonologically related conditions with overlap in handshape-only, location-only, or both handshape and location. Irrespective of the type of phonological overlap, we found that target signs elicited larger amplitude N400s in the semantic task compared to the repetition detection task (cf. Fig. 3). This is the first report of task demands influencing N400 amplitude elicited by sign stimuli, but this finding mirrors a broader body of literature demonstrating similar task effects for words (e.g., Chwilla et al., 1995; Meade et al., 2019). The larger amplitude N400 in the semantic task suggests that our task manipulation was successful at influencing processing and may indicate that participants were engaging comparatively deeper lexico-semantic processing when performing semantic categorization relative to repetition detection. Longer reaction times for probe items (i.e., “go” responses) in the semantic task than for the repetition task further support this contention.

The type of processing required by the task also modulated the magnitude and direction of the phonological N400 priming effects, in different ways depending on the specific parameters that were manipulated. When the prime and target overlapped in both handshape and location or handshape only, targets preceded by related primes elicited smaller N400s overall than those preceded by unrelated primes (i.e., standard priming). Whereas the two-parameter priming effect was robust and statistically similar across tasks, the handshape priming effect was only present in the repetition detection task. The most striking impact of the task manipulation was the significant interaction observed for location-only priming. We replicated the “reversed” phonological priming effect (i.e., smaller amplitude N400s for targets in unrelated pairs compared to location-related pairs) previously reported in a lexical decision task by Gutiérrez and colleagues (2012) in our semantic task, although it did not reach significance. In contrast, the priming effect in the repetition detection task trended in the standard direction (i.e., smaller amplitude N400s for targets in location-related pairs compared to unrelated pairs). In what follows, we outline the implications that these results have for our understanding of the processes that underlie sign recognition at three levels: activation of sublexical representations, lexical competition, and top-down attention.

The first factor that we consider is pre-activation of shared sublexical representations. Processing a prime sign involves activation of the sub-lexical parameters of which it is composed. Interactive-activation models of word processing further assume that activation feeds back from all co-activated lexical representations (i.e., the prime sign and its neighbors) to reinforce activation of these sublexical units (e.g., McClelland & Elman, 1986; McClelland & Rumelhart, 1981). The 1300 ms SOA that we used provided ample time for such reverberation to occur before the target sign was presented. Thus, when the target was composed of the same sublexical parameters as the prime sign (i.e., in the related conditions), this sublexical pre-activation and reverberation should have facilitated target processing, yielding smaller amplitude negativities. If we assume that sublexical facilitation is approximately proportional to the number of shared parameters, then this would explain why the two-parameter priming was particularly strong and robust across tasks.

Unique to sign languages is the consideration of how different sublexical parameters influence recognition and lexical organization. As discussed in the Introduction, Caselli and Cohen-Goldberg (2014) have argued that the reversal in the direction of priming patterns observed for handshape-only versus location-only overlap arises at the sublexical level. They were able to simulate the reversal when information about either the respective timing or sublexical frequency of these two parameters was introduced into their computational model. If it is true that the dissociation in the direction of priming effects for these two parameters occurs at the sublexical level, then we would have expected qualitatively similar patterns across the two tasks, which is not what we found. The relative timing and frequency of the location and handshape parameters remained constant across the two tasks and therefore cannot account for why the location priming effect trended in different directions across tasks.

In contrast, lexical competition can be used to explain the different patterns across tasks. The two tasks differed in the extent to which they required participants to identify a specific lexical representation. Presumably, the repetition detection task could be done purely on the basis of surface-level perceptual or sublexical similarity and could even be accomplished by participants who had no knowledge of ASL. That is not to say that participants were not activating lexico-semantic knowledge, but rather that the task did not require them to suppress co-activated neighbors and home in on one specific lexico-semantic representation. In contrast, the semantic categorization task required participants to identify the specific lexico-semantic representations of the targets and to evaluate their category membership. Arguably, the increase in lexical competition that was leveraged to identify single signs in the semantic categorization task could have been what yielded the trend for “reversed” location priming. If processing of the prime sign involved lateral inhibition of the lexico-semantic representations of co-activated neighbors, then processing effort for targets in the location-related condition should increase relative to phonologically unrelated target signs that do not undergo such inhibition (see also Carreiras et al., 2008; Gutiérrez et al., 2012). This process would be somewhat analogous to the lexical competition among neighboring words that is a prominent feature of interactive-activation models (e.g., McClelland & Elman, 1986; Meade, Grainger, Midgley, Emmorey, & Holcomb, 2018). With respect to signed languages, our results indicate that location is a critical parameter in determining which lexico-semantic representations engage in this competition (see also Gutiérrez et al., 2012). The key in determining the size and direction of the net priming effect is the relative strength of sublexical facilitation and this lexical competition.

As alluded to above, this notion of lexical competition in the interactive-activation framework is closely connected with the notion of neighborhood density, but there are important distinctions. Assuming that lexical competition is involved in driving differences in processing between lexical items with few versus many neighbors (e.g., Meade et al., 2019), then by extension location would also be critical for defining neighborhood density in signed languages (see also Carreiras et al., 2008). However, it is unlikely that neighborhood density *per se* contributed to the effects that we observed since the same target items (i.e., with the same neighborhood densities) appeared across the related and unrelated conditions in the two tasks. In other words, the nature of the connections between phonologically related signs in the lexical network, and not the number of connections, contributed to our results.

The third and final factor that we consider is top-down attention, which we assume is also largely regulated by task demands. In the repetition detection task, the judgment was related to the form of the sign and likely biased participants to attend to form-level features. This reasoning is supported by the presence of a late positivity for targets in related pairs that can been seen in Fig. 3. The late P300-like positivity was especially prominent in the repetition task and appears to have increased the size of the priming effect.^2^ This pattern could be interpreted to suggest that participants initially mistook the strong resemblance between related primes and targets as repetition probe items. In contrast, the form resemblance would not lead participants to consider related items as probes in the semantic categorization task. A similar pattern is revealed in the false alarm data: related HS + LOC targets were especially likely to elicit false alarm button presses in the repetition detection task, but this was not the case in the semantic categorization task. The general idea that selective attention modulates phonological priming is supported by auditory word recognition studies. For example, Yoncheva and colleagues have found that ERP rhyme priming effects were essentially limited to phonological tasks (2013, 2014). Given the evidence that sign phonological priming persists in semantic tasks, both in the present study and in the literature (e.g., Gutiérrez et al., 2012; Meade, Lee, et al., 2018), it would appear that some subset of the processes that underlie phonological priming may be automatic (i.e., occur irrespective of task). However, the comparison across tasks here highlights that explicitly orienting participants’ attention toward the form of the signs in the repetition detection task further enhances the size of the phonological priming effect by including a strategic component.

In conclusion, the present results begin to define the role that each phonological parameter plays in sign recognition and solidify the special status of the location parameter. The comparison of location priming across tasks is more consistent with the notion that location is differentiated from the other parameters at the lexical, rather than sublexical, level. If the priming effect was due to sublexical frequency or the early availability of location information, then we would expect qualitatively similar patterns irrespective of task. Rather, the demands of the semantic categorization task – specifically the need to engage lexical competition to identify a single target sign – appeared to overwhelm sublexical facilitation and reverse the direction of the priming effect across tasks. Overall, these results suggest that the architecture of the sign lexicon and the process of recognizing single signs are similar to those for spoken language. They also emphasize the importance of the empirical study of the sign language lexicon in order to understand how each of the multiple sublexical parameters that compose signs are processed.

## Figures and Tables

**Table 1:** Artifact rejection rates per condition [mean (SD)]

	HS + LOC		LOC		HS	
	Related	Unrelated	Related	Unrelated	Related	Unrelated
Repetition Detection	3.9 (2.7)	4.2 (2.8)	3.8 (2.9)	4.5 (2.7)	4.7 (3.4)	5.0 (2.6)
Semantic Categorization	3.5 (3.1)	3.9 (3.8)	2.9 (2.9)	3.0 (2.0)	3.3 (2.4)	3.2 (3.5)

**Table 2:** False alarm rates for critical trials per condition [mean (SD)]

	HS + LOC		LOC		HS	
	Related	Unrelated	Related	Unrelated	Related	Unrelated
Repetition Detection	1.1 (1.5)	0.2 (0.4)	0.0 (0.0)	0.2 (0.4)	0.4 (0.5)	0.2 (0.4)
Semantic Categorization	0.9 (1.1)	1.4 (1.5)	2.0 (1.4)	3.0 (2.2)	2.5 (2.0)	1.7 (1.2)

Note: There were 46 trials per condition.

## Footnotes

1 The data related to this project are available at https://osf.io/w7pj4/. A table with all statistical tests for the N400 window is provided in the Supplementary Materials. As suggested by a reviewer and keeping in line with our previous ASL phonological priming study (Meade, Lee, et al., 2018), we have also included the full set of results for mean amplitude within an N300 window (200–400 ms).

2 A significant interaction between Prime and Task in this later window (600–800 ms) confirmed that the priming effect was larger overall in the repetition detection task relative to the semantic task, *F*(1,19) = 37.27, *p* < .001, $\eta_{\text{p}}^{2}=0.66$.
